# *SSTR2* promoter hypermethylation is associated with the risk and progression of laryngeal squamous cell carcinoma in males

**DOI:** 10.1186/s13000-016-0461-y

**Published:** 2016-01-22

**Authors:** Zhisen Shen, Xiaoying Chen, Qun Li, Chongchang Zhou, Jinyun Li, Huadan Ye, Shiwei Duan

**Affiliations:** Department of Otorhinolaryngology (Head and Neck Surgery), Lihuili Hospital of Ningbo University, Ningbo, Zhejiang 315040 China; Zhejiang Provincial Key Laboratory of Pathophysiology, School of Medicine, Ningbo University, Ningbo, Zhejiang 315211 China

**Keywords:** Laryngeal carcinoma, *SSTR2*, DNA methylation, Male

## Abstract

**Background:**

Somatostatin receptor 2 (*SSTR2*) encodes somatostatin receptor that can inhibit the cell proliferation of solid tumors. Promoter hypermethylation is likely to silence the expression of *SSTR2*. The goal of our study was to investigate the association between *SSTR2* promoter methylation and the risk and progression of laryngeal carcinoma.

**Methods:**

In the current study, tumor tissues and their adjacent non-tumor tissues were collected from a total of 87 laryngeal squamous cell carcinoma (LSCC) male patients. DNA methylation levels of nine *SSTR2* promoter CpGs were measured using the bisulphite pyrosequencing technology.

**Results:**

Our results revealed that there was a significantly increased *SSTR2* promoter methylation in LSCC tissues than in their adjacent non-cancerous tissues (adjusted *P* = 0.003). Breakdown analysis by age indicated that the significant association was mainly contributed by patients younger than 60 (adjusted *P* = 0.039) but not in patients older than 60. Meanwhile, the significant association was observed in the patients with moderately (adjusted *P* = 0.037) and well differentiated tissues (adjusted *P* = 0.028), as well as the patients with histological stage IV (adjusted *P* = 0.031). Multivariate Cox analysis suggested that *SSTR2* promoter methylation was an independent prognostic factor of LSCC (HR = 1.127, 95 % CI = 1.034–1.228).

**Conclusions:**

In conclusion, *SSTR2* promoter hypermethylation might be associated with the risk and progression of LSCC in males.

## Background

Laryngeal cancer is a devastating malignancy of head and neck, and its incidence and mortality rates are increasing recently [1]. Despite improvement of oncological and surgical treatments over the last 20 years, 5-year survival rates of laryngeal cancer remained poor since the middle of 1980s [2, 3]. More than 90 % of laryngeal cancer has been pathologically identified as laryngeal squamous cell carcinoma (LSCC) [1]. According to the Cancer Facts & Figures 2015 data, the majority of laryngeal cancer patients are males.

Currently, total laryngectomy and postoperative radiotherapy are the most common treatments for LSCC [4]. Due to serious impairment in laryngeal function and low quality life that brings for patients, the exact molecular pathogenesis of LSCC is still urgently needed to be explored. It is hypothesized that LSCC may result from the interactions of many complex factors, including environmental factors (smoking, alcohol consumption, air pollution, and infection) and genetic factors [5]. Meanwhile, accumulating studies suggest that there are numerous aberrant epigenetic modifications in laryngeal cancer [6].

Somatostatin (SST) is an important peptide in the regulation of cell secretion and proliferation [7]. It operates through engagement on a family of 5 transmembrane G-protein coupled somatostatin receptors (SSTRs 1–5) [8]. Somatostatins have a function of anti-proliferation, pro-apoptosis and anti-migration, and thus play a role in the suppression of tumor growth. Hypermethylation of *SSTR1* gene along with reduced expression was found in head and neck squamous cell carcinoma [9], and the reversed *SSTR5* methylation was shown to up-regulate *SSTR5* mRNA expression in prostate cancer [10]. SSTR2 is widely distributed and it is responsible for the anti-proliferative effect of somatostatin and its analogs in vitro and in vivo [11]. SSTR2 protein levels were significantly lower in the malignant larynx than the pre-malignant larynx [12]. However, there was a lack of association study between *SSTR2* methylation and laryngeal cancer.

Epigenetic modifications are crucial for tumorigenesis [13]. In mammalian cells, DNA methylation, as one of the most common modifications, occurs mainly at the C5 position of cytosine-phosphate-guanine (CpG) dinucleotides [14]. Genes with aberrant DNA methylation have been shown with great potential in the early detection and prognosis of cancers [15–18]. In light of previous findings, we aimed to test whether *SSTR2* promoter methylation contributed to the pathogenesis of laryngeal cancer.

## Methods

### Tissues samples

**Fig. 1:**
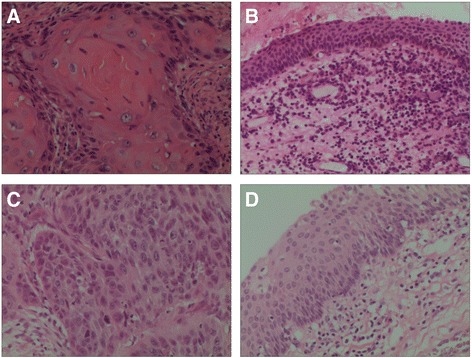
Histopathology of test specimens. **a** Well differentiated tumor tissues of LSCC (x200). **b** Well differentiated non-tumor tissues of LSCC (x200). **c** Poorly differentiated tumor tissues of LSCC (x200). **d** Poorly differentiated non-tumor tissues of LSCC (x200)

All the tumors and their paired adjacent non-tumor tissues were collected from 87 male patients ranging from 40 to 86 years old. All the tissues were postoperative laryngeal specimens obtained from the LSCC male patients. There were 73 smokers and 14 non-smokers. And there were 40 well differentiated cases (Figures 1a and n), 32 moderately differentiated cases, and 15 poorly differentiated cases (Figures 1c and D) according to their pathologic diagnoses. And there were 25 stage I, 14 stage II, 10 stage III, and 38 stage IV patients. There were 43 cases aged younger than 60 years, 33 cases aged 60–70 years, and 11 cases aged older than 70 years. Overall survival (OS) data was recorded from 54 out of 87 patients between June 2010 and August 2015. The controls were the non-cancerous adjacent tissues that were obtained from at least 5 cm outside the edge of tumors, although they might not accurately represent non-cancerous control tissues. Generally, total laryngectomy is the most common treatment for laryngeal cancer patients. Moreover, surgery-only laryngeal cancer patients had significantly better survival than those with radiotherapy or chemotherapy [19]. In addition, chemoradiotherapy was shown to affect the DNA methylation level [20]. Therefore, we excluded the patients with radiotherapy or chemotherapy in our study. All the specimens were obtained fresh and stored at −80 °C. None of the patients had a history of hereditary cancer. All the participants in the study have signed the informed consent forms. Permission was also obtained from the local ethics committee to access the pathology archives at Department of Otolaryngology at Ningbo Lihuili Hospital, Ningbo, Zhejiang, China.

### Bisulfite pyrosequencing methylation assay and primer sequences

Genomic DNA extraction from tumor samples and subsequent quantification procedures were as described before [21]. The details of DNA bisulfite conversion were as described previously [21]. The sequences were 5’-AGGGTAGAGGAGTTAGGAATTT-3’ for the forward primer, 5’-Biotin-ACCCCTCACCTTTACTTTTC-3’ for the reverse primer, and 5’-ACCCAACCACTATCCC-3’ for the sequencing primer.

### Statistical analyses

Statistical analyses were performed using SPSS v18.0 (SPSS Inc., Chicago, IL). Data was expressed as mean ± standard deviation (SD). Analysis of variance (ANOVA) was used to evaluate the association between the risk factors (including age, smoking behavior, histological differentiation and clinical stage) and their relative methylation rate difference. Paired sample *T*-test and non-parametric test were performed to compare *SSTR2* methylation levels between cancer tissues and adjacent tissues. All the *P* values were adjusted by logistic regression. Multivariate Cox proportional hazard models were used to calculate hazard ratios (HR) and the corresponding 95 % confidence intervals (95 % CI). A two-tailed *P* < 0.05 was considered to be significant. Figures were drawn using GraphPad Prism 6 software (GraphPad Inc., San Diego, CA).

## Results

**Fig. 2:**
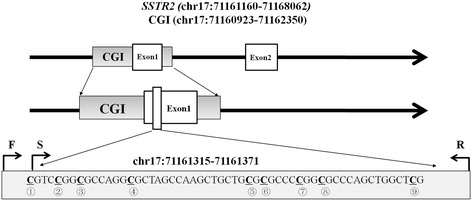
The nine tested CpG dinucleotides in *SSTR2* gene ^a^. ^a^: F stands for forward primer; S stands for sequencing primer; R stands for reverse primer

In the current study, we have tested nine CpG dinucleotides in the *SSTR2* CpG island (CGI, chr17:71160923–71162350) to measure *SSTR2* methylation levels using the bisulfite pyrosequencing technology (Figure 2). The raw methylation data of nine CGs on *SSTR2* gene promoter were presented in Supplemental Dataset 1. Using the mean methylation level, we preformed the association study between 87 male tumor tissues and their adjacent non-tumor tissues. In addition, the differences in *SSTR2* methylation level were not statistically associated with age, smoking behavior, histological differentiation or clinical stage in tumor tissues or paired tissues (all adjusted *P* > 0.05, data not shown).

**Fig. 3:**
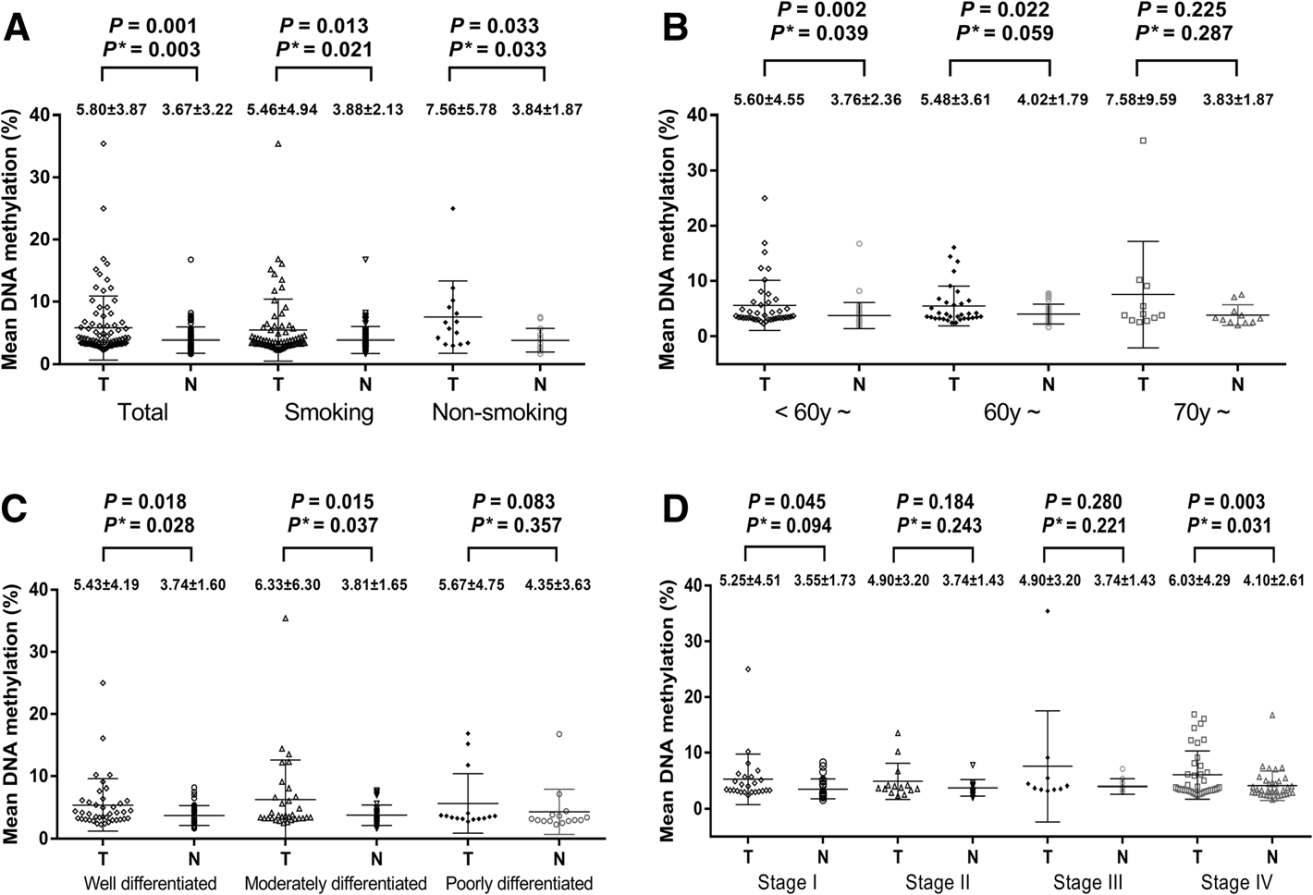
Comparisons of mean methylation level of *SSTR2* gene between tumor tissues and paired adjacent non-tumor tissues by (**a**) total and smoking behavior, (**b**) age, (**c**) differentiation and (**d**) clinical stage ^a^. ^a:^ T stands for tumor tissues; N stands for non-tumor tissues. P*: adjusted by age, smoking behavior, differentiation and clinical stage

Our results showed that *SSTR2* promoter methylation levels in cancer tissues were significantly higher than in the paired non-tumor tissues (Figure 3a, 5.80 ± 3.87 % versus 3.67 ± 3.22 %, *P* = 0.001, adjusted *P* = 0.003). Subgroup analysis by the smoking status showed that *SSTR2* promoter hypermethylation was associated with the risk of laryngeal cancer in the patients with and without smoking behaviors (Figure 3a, smokers: 5.46 ± 4.94 % versus 3.88 ± 2.13 %, *P* = 0.013, adjusted *P* = 0.021; non-smokers: 7.56 ± 5.78 versus 3.87 ± 1.87, *P* = 0.033, adjusted *P* = 0.033). Further subgroup analysis by age showed that the patients aged <60 years old showed a statistically higher methylation in cancer tissues than in paired non-tumor tissues, but this association could not be found in those older than 60 years old (Figure 3b, <60y: adjusted *P* = 0.039; 60y~: adjusted *P* = 0.059; 70y~: adjusted *P* = 0.287).

DNA promoter methylation is associated with clinicopathological characteristics, such as differentiation and clinical stages [22, 23]. Therefore, we conducted a histological differentiation-based comparison between tumor and non-tumor tissues. And our results showed a significant higher methylation in the well differentiated (Figure 3C, adjusted *P* = 0.028) and moderately differentiated LSCC (adjusted *P* = 0.037) compared with their adjacent tissues, but the significant association was not found in poorly differentiated LSCC compared with the controls (adjusted *P* = 0.357). In addition, clinical stage-based subgroup analysis showed a significant hypermethylation in stage IV (Figure 3D, adjusted *P* = 0.031), but not in other stages (stage I: adjusted *P* = 0.094; stage II: adjusted *P* = 0.243; stage III: adjusted *P* = 0.221).

In order to investigate the association between SSTR2 methylation level and OS in LSCC patients, we performed a multivariate Cox proportional hazard analysis by adjusting age, stage, differentiation and smoking behavior. As shown in Table 1, the poor OS of LSCC patients was found to be significantly associated with *SSTR2* promoter hypermethylation (HR = 1.127, 95 % CI = 1.034–1.228).

**Table 1:** Multivariate Cox proportional hazards analysis in 54 LSCC patients ^a^

Characteristics	N	*P* value	HR	95 % CI
Age	54	0.821	0.992	0.925–1.064
Stage				
Stage I (Ref)	15	-	1.000	-
Stage II	8	0.106	6.794	0.665–69.416
Stage III	7	1.865	4.865	0.408–57.962
Stage IV	24	0.016	13.630	1.636–113.539
Differentiation				
Well (Ref)	21	-	1.000	-
Moderated	21	0.706	1.267	0.370–4.344
Poorly	12	0.555	1.401	0.457–4.299
Smoking behavior				
No (Ref)	7	-	1.000	-
Yes	47	0.271	2.699	0.461–15.813
*SSTR2* methylation	54	0.006	1.127	1.034–1.228

^a^: the overall survival information of 33 LSCC patients was unavailable; Ref: reference category; HR: hazard ratio

## Discussion

Our study firstly investigated the role of *SSTR2* promoter methylation in the risk and progression of LSCC. A significantly increased *SSTR2* promoter methylation was found in LSCC tissues than non-cancerous tissues. Meanwhile, multivariate Cox analysis suggested that *SSTR2* promoter methylation was an independent prognostic factor of LSCC. Our results provided a clue for further studies on the role of *SSTR2* in laryngeal carcinogenesis, and future studies were needed to confirm its potential as a biomarker for early diagnosis, therapy and prognosis of laryngeal cancer.

Previous laryngeal cancer study has shown that the overall pattern of SSTRs expression is with high levels of SSTR1, “loss” of SSTR2 and intermediate levels of SSTR5 [24]. For other SSTR members, there was very little expression of SSTR3 detected in benign and pre-malignant specimens and malignant laryngeal tumors. Meanwhile, a variable degree of low to moderate levels of SSTR4 expression was detected across these three groups [12].

DNA hypermethylation in the promoter of tumor suppressor genes (TSGs) inhibits transcriptional initiation and results in the silencing of TSGs. Hypermethylated promoters of tumor suppressor genes (*CHD5* [25], *CHFR* [26], *PTEN* [27], *FHIT* [28], *CDKN2B* [29], *APC* [29], *DAPK1* [29]) have been shown as an important epigenetic mechanism in LSCC. Moreover, accumulating studies have reported other aberrantly hypermethylated genes in LSCC, including *CBY* [30], *IGFBP-rP1* [31], and *MYCT1* [6]. Here, we provided another hypermethylated gene (*SSTR2*) in LSCC. Alternation of *SSTR2* promoter methylation level in LSCC is not fully understood. Here, we observed a significantly increased *SSTR2* promoter methylation in cancerous tissues than in their adjacent tissues, especially in the stage IV LSCC patients. Besides, we found that *SSTR2* promoter hypermethylation could predict a poor OS of LSCC. Taken together, these supported that the “loss” of *SSTR2* expression in laryngeal carcinoma, especially in the late stage, might be due to a higher methylation of *SSTR2*. Although mRNA levels were not determined in these specimens, our results tended to be compatible with the previous results of the previous laryngeal cancer study [32].

According to the Cancer Facts & Figures 2015 data (http://www.cancer.org/research/cancerfactsstatistics/cancerfactsfigures2015/index), most the laryngeal cancer patients are males. In the current study, we only recruited very few female samples. In order to get rid of heterogeneity by gender, we decided to focus on the study in males for the moment. Besides, we have performed smoking status, age, histological differentiation and stage subgroups analyses.

Age plays an important role in the carcinogenesis [33]. Interestingly, the significantly higher methylation was found in the younger population (<60 years), which might provide a potentially age-specific biomarker of laryngeal cancer.

SSTR2 is widely expressed in normal tissues, and it is a negative regulator of cell proliferation in human tumorigenesis [34]. Increased *SSTR2* expression led to strong up-regulation of cyclin-dependent kinase inhibitor p16, which then inhibited tumor cell cycle progression from G1 to S phase [35]. In addition, SSTR2 deficiency may contribute to the development of tissue invasion and metastasis process [36]. Lower mRNA levels of *SSTR2* were expressed in the metastases from prostate cancers than in primary prostate cancers, meanwhile, decreased SSTR2 could predict poor prognosis of prostate cancer [35]. Our breakdown analysis showed a significant association of *SSTR2* methylation with LSCC risk in moderately and well differentiated LSCC patients but not in the poorly differentiated patients. Additionally, there was a significant difference could be found in stage IV group, even adjusted by risk factors of age and smoking. Our results provided a hypothesis that the *SSTR2* methylation might be involved in the metastasis and invasion of laryngeal cancer.

However, there were limitations in the current research to be considered. Firstly, our study was involved with 87 LSCC cancerous and 87 non-cancerous tissues. Power analysis showed 90.8 % power for overall test and 15.1–100.0 % power for the subgroup analyses by differentiation, histological stages of cancerous tissues and age. Thus, our observations in the total samples were reliable, however, the negative results in the subgroup analyses might need to be confirmed with larger sample size in the future. Secondly, the professional background was a potential risk factor in laryngeal disease. A previous study has reported that professional voice users such as singers may be susceptible to laryngeal disease [37]. Unfortunately, we didn’t collect the professional background of patients.

## Conclusions

This study indicated that *SSTR2* promoter hypermethylation was associated with the risk and progression of laryngeal cancer in males. These findings provided clues for further studies on the role of *SSTR2* in laryngeal carcinogenesis and its potential as a biomarker for early diagnosis, therapy and prognosis of laryngeal cancer.

## Abbreviations

CGI: CpG island
CpG: cytosine-phosphate-guanine
DNA: deoxyribonucleic acid
LSCC: laryngeal squamous cell carcinoma
LSCC: laryngeal squamous cell carcinoma
*SSTR2*: aomatostatin receptor 2
